# Proton Magnetic Resonance Spectroscopy at 3.0T in Rabbit With VX2 Liver Cancer: Diagnostic Efficacy and Correlations With Tumor Size

**DOI:** 10.3389/fonc.2022.846308

**Published:** 2022-03-31

**Authors:** Ruikun Liao, Zhuoyue Tang, Xiaojiao Li, Liang Lv, Chao Yang, Hua Xiong, Bi Zhou, Jiayi Yu, Dan Zhang

**Affiliations:** ^1^Department of Radiology, Chongqing General Hospital, Chongqing, China; ^2^Molecular and Functional Imaging Laboratory, Chongqing General Hospital, Chongqing, China

**Keywords:** magnetic resonance, proton magnetic resonance spectroscopy, tumor size, choline peak, liver cancer

## Abstract

**Purposes:**

The purpose of this study was to explore the diagnostic performance of Cho peak area (Cho Are), Cho peak amplitude (Cho Amp), and the combined approach (Cho Are_Amp) in detecting rabbit VX2 liver cancer at the early stage *via* hydrogen-1 proton magnetic resonance spectroscopy (^1^H-MRS), as well as the correlations between Cho Are, Cho Amp, and tumor parameters like diameter and volume.

**Methods:**

Conventional magnetic resonance imaging (MRI) and MRS were performed to scan the VX2 liver cancer in rabbit. The tumor diameter was measured on T2-weighted imaging (T2WI), and the tumor volume was accordingly calculated. Cho Are and Cho Amp were obtained from MRS. The diagnostic performance of Cho Are, Cho Amp, and Cho Are_Amp was assessed by a receiver operating characteristic (ROC) curve and area under ROC curve (AUC), whereas specificity and sensitivity were calculated by the maximum Youden’s index. Spearman’s correlation analysis was performed to evaluate the relevance between tumor parameters (diameter, volume) and radiological indexes (Cho Are, Cho Amp).

**Results:**

ROC curve analysis showed that Cho Amp, Cho Are, and Cho Are_Amp were effective in diagnosing VX2 liver cancer. The AUC of Cho Amp was 0.883, and the specificity and sensitivity were 0.944 and 0.722, respectively (*p* < 0.001). The AUC of Cho Are was 0.807, and the specificity and sensitivity were 0.778 and 0.833, respectively (*p* < 0.05). The AUC of Cho Are_Amp was 0.892, and the specificity and sensitivity were 0.833 and 0.833, respectively (*p* < 0.001). Cho Are and Cho Amp exhibited a high positive correlation with tumor diameter and tumor volume, among which Cho Amp demonstrated better correlations to tumor diameter and tumor volume (r = 0.956 and 0.946) than that of Cho Are (r = 0.787 and 0.794). A high positive correlation was detected between Cho Are and Cho Amp (r = 0.787), as well as tumor diameter and tumor volume (r = 0.965).

**Conclusion:**

Cho Are_Amp can be used as an effective tool in diagnosing early-stage VX2 liver cancer with satisfied diagnostic accuracy. Cho Are and Cho Amp were positively correlated with tumor volume and tumor diameter. The results of this study provide further evidence that Cho Amp and Cho Are_Amp of MRS could aid in the early diagnosis of liver cancer.

## Introduction

Liver cancer is one of the most common malignant tumors worldwide. The indolent and asymptomatic nature at the early stage frequently delays the clinical diagnosis, contributing to poor clinical prognosis. Therefore, early and specific diagnosis is critical to improving clinical outcomes (1, 2).

Imaging techniques, such as MRI, were pivotal in clinical liver cancer diagnosis. Despite relatively high diagnostic efficacy in advanced liver cancer, some limitations were reflected in early-stage cancer detection in conventional MRI (3). ^1^H-MRS is an imaging technology for quantitative and semiquantitative analyses of metabolites in living organs based on hydrogen proton chemical shift effect. As a non-invasive and non-radiative approach, the diagnostic value of ^1^H-MRS is clinically tested in organs with less mobility, such as brain, prostate, and breast (3, 4). However, its application in organs like liver, interfered by respiration and intestinal movement, remains exploratory (5, 6).

It was well documented that the liver serves as a major organ in the bio-transformation and metabolism of a variety of substances. During the early-stage hepato-tumorigenic process, choline compounds represented the most significant enhancive substance across metabolites, and choline compounds increased exponentially with the tumor growth (7–9). As the Cho peak of MRS could effectively reveal the change of choline compounds, it could serve as a reasonable strategy for quantitative and semiquantitative diagnoses of early-stage liver cancer. The measurement accuracy of the Cho peak rested with the spectral line stability, a parameter affected by multiple factors, including respiratory movement, magnetic field intensity, magnetic field uniformity, and full width at half maximum (10). Correspondingly, Cho Amp was proposed as an alternative index with improved spectral line stability and diagnostic efficiency together and being less affected by the magnetic field uniformity (9–11).

In this study, we successfully established the rabbit model of VX2 orthotopic liver cancer and then utilized a combined approach of anesthesia and respiratory-gated navigation to minimize the interference from respiratory and intestinal movement. We performed an MRS examination and detected the change in both Cho Are and Cho Amp. We further evaluated the diagnostic efficiency of Cho Are, Cho Amp, and their combination, as well as the correlations within and between radiological indexes (Cho Are, Cho Amp) and tumor parameters (diameter, volume).

## Methods

### Establishment of Animal Models

This study included 18 New Zealand white rabbits (age, 2–3 months, gender, male and female, weight, 2.0–3.0 kg). They were purchased from the animal experiment center of Chongqing Medical University and routinely raised in the animal experiment center. The VX2 tumor cell line was purchased from the Institute of Biomedical Engineering Ultrasound, Chongqing Medical University. All the protocols were approved by the Laboratory Animal Welfare and Ethics of the Third Military Medical University (SYXK-PLA-20120031). The rabbit thigh skin was prepared, the VX2 tumor cell line was implanted under the skin of the inner left thigh of the rabbit, the tumor growth was observed intermittently, and the tumor was cut out when its diameter was greater than 3 cm. After washing with normal saline, the white tumor parenchyma was cut into small pieces of 1–3 mm^3^ for standby. Sodium pentobarbital (0.1–0.2 ml) was intermittently anesthetized through the vein of the rabbit ear edge, and the tumor tissue block was punctured and inoculated under open vision. It was implanted in the deep surface of the middle lobe of the liver. Gelatin sponge was given to prevent implant metastasis when the needle was withdrawn. The abdomen was closed after gauze hemostasis. Antibiotics were routinely used for 3 days to prevent infection.

### Magnetic Resonance Imaging and Magnetic Resonance Spectroscopy

A previous study reported that the early stage (parenchymal stage) of rabbit VX2 tumor is 2–3 weeks after the tumor was implanted (12). Therefore, the magnetic resonance scanning was performed on rabbits 2 weeks after the tumor was implanted in our study. Fasting and water deprivation were performed 4–6 h before scanning. Under continuous anesthesia (3% pentobarbital sodium, 40 mg/ml), the interference of respiratory and intestinal movement artifacts was reduced *via* Siemens respiratory gating technology. A routine MRI and MRS scanning of rabbits was completed by 3.0T superconducting magnetic resonance (Siemens Verio, 8-Channel, surface coil). Before MRS scanning, an automatic pre-scanning was performed to complete homogenization and water suppression. After the pre-scanning, the T2WI sequence was used as ^1^H-MRS positioning. The MR parameters are as follows. (I) The conventional MR scanning included axial T1-weighted spin echo: time of repetition (TR)/time of echo (TE): 450 ms/9.0 ms, slice thickness: 5.0 mm, field of view (FOV): 210 × 210 mm, matrix: 256 × 192, and average: 2; axial, sagittal, coronal T2-weighted fast spin echo: TR/TE: 2,800 ms/95 ms, slice thickness: 5.0 mm, FOV: 210 × 210 mm, matrix: 256 × 192, and average: 4; axial T1-weighted imaging (T1WI)-Vibe 3-dimensional volume interpolation fast disturbing phase gradient echo: TR/TE: 4.6 ms/1.7 ms, slice thickness: 2.0 mm, FOV: 210 × 210 mm, matrix: 320 × 272, and average: 2. (II) MRS: TR/TE: 2,000 ms/135 ms, FOV: 210 × 210 mm, matrix: 1 × 1, flip angle: 90°, and average: 130. The size of the MRS voxel is 2 cm × 2 cm × 1.8 cm, which was placed in the tumor parenchyma and the left lobe of the liver next to the middle hepatic vein, away from the edge of the liver, trying to avoid large blood vessels, diaphragmatic surface, and intrahepatic bile duct.

### Image Processing and Analysis

The Siemens MRI self-contained post-processing workstation (Syngo B19) was used to measure the size on axial and coronal T2WI images. In order to reduce the error, the data measurement was jointly completed by two senior radiologists and their consistency was tested. The size of the VX2 tumor was measured at the largest level, and the average size was taken after three measurements. In this study, the adopted calculation method of the tumor volume formula was [L × W × H ×Π/6] according to the experimental results of the previous study (13).

The water suppression, zero filling, phase correction, and baseline correction were performed on the raw data through Siemens magnetic resonance image post-processing software after MRS was completed. Meanwhile, the Cho peak area and Cho peak amplitude were recorded at 3.22 ppm in MRS. The spectra with obvious baseline deformation, excessive noise, and unrecognizable choline peak were not included in the analysis range.

### Histology Analysis

All rabbits were killed by intravenous injection of excessive pentobarbital sodium after routine MR and MRS examination. After the whole liver was removed and fixed, the tumor tissue and liver tissue were separated from it. The section direction should be consistent with the MR scanning coronal direction as far as possible, and the tissue sections should be embedded in paraffin. The sections were stained with hematoxylin and eosin (H&E), and the tumor tissue and liver tissue were observed under the microscope.

### Statistical Methods

The data were analyzed by SPSS 22.0 software (IBM, Chicago). ICC coefficient consistency evaluation was used to detect the consistency of tumor diameter and tumor volume measurement. To determine the correlations between tumor diameter, tumor volume, and Cho Are and Cho Amp, Spearman correlation analysis was performed with an r value of 0.71–1.00 being considered highly correlated, 0.40–0.70 being moderately correlated, and ≤0.40 being poorly correlated. The diagnostic performance of Cho Are, Cho Amp, and Cho Are combined with Cho Amp (Cho Are_Amp) in liver cancer was tested *via* the ROC curve analysis. The cutoff values were determined by calculating the maximal Youden index. A *p* value of less than 0.05 was considered statistically significant.

## Results

### MRI and MRS

**Figure 1:**
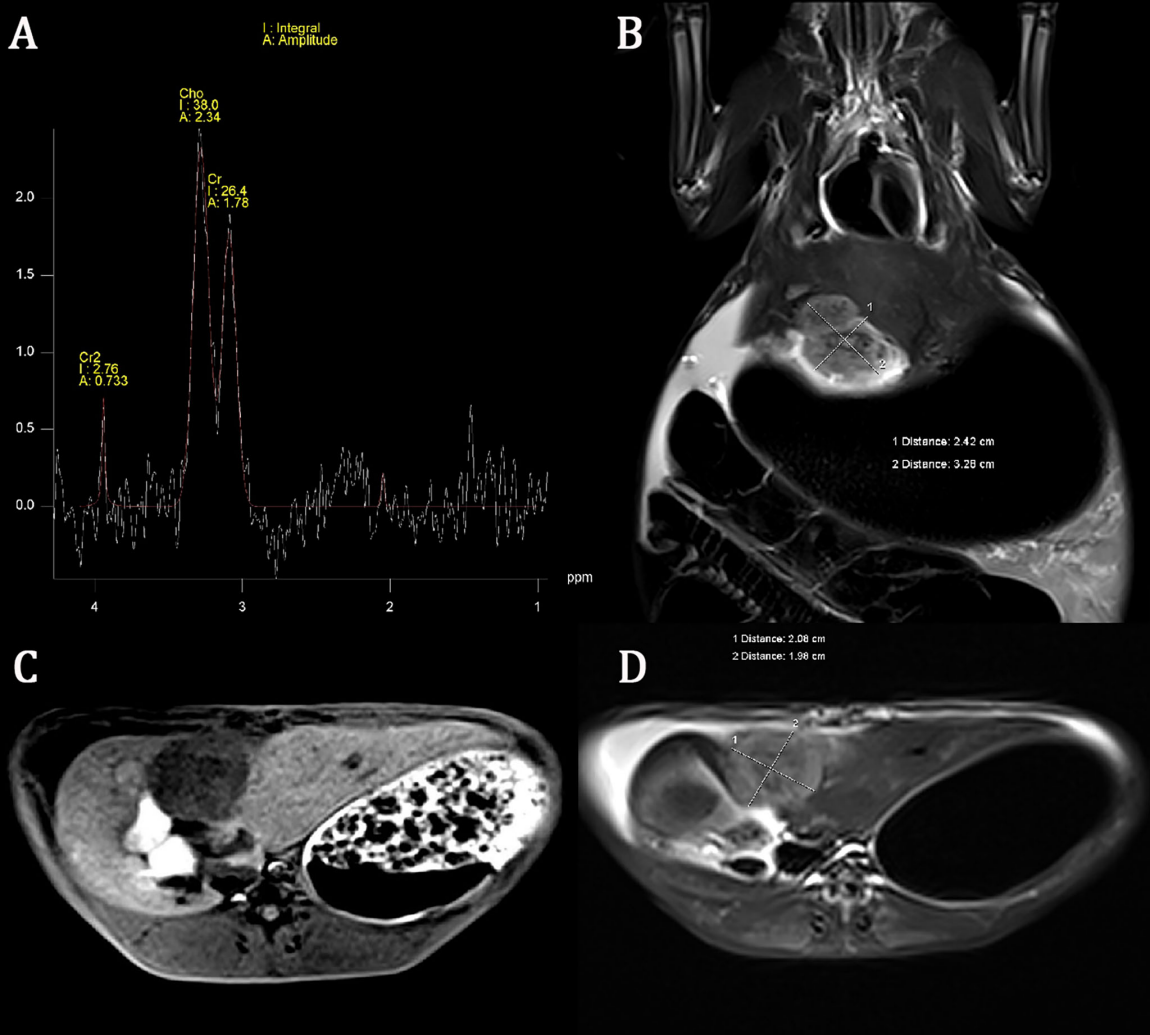
Conventional magnetic resonance imaging (MRI) sequence and hydrogen-1 proton magnetic resonance spectroscopy (^1^H-MRS) of liver cancer. The choline (Cho) peak at 3.22 ppm is significantly increased in liver VX2 tumor **(A)**. The axial and coronal images of the tumor showed hypointensity on T1-weighted imaging (T1WI) and slightly hyperintensity on T2-weighted imaging (T2WI) **(B–D)**.

All rabbits survived after tumor implantation 2 weeks later, and the conventional MRI and MRS examination was completed successfully. The image of the conventional sequence was clear and recognizable, the tumor boundary was clear, and the tumor showed hypointensity on T1WI and slightly hyperintensity on T2WI. The spectral lines of MRS data corrected by post-processing software were stable and without obvious overlapping peaks. All spectral lines showed a high Cho peak at 3.22 ppm. Cho Are and Cho Amp could be used for spectral analysis, as shown in **Figure 1**.

### Tumor Size Measurement and Correlation

**Figure 2:**
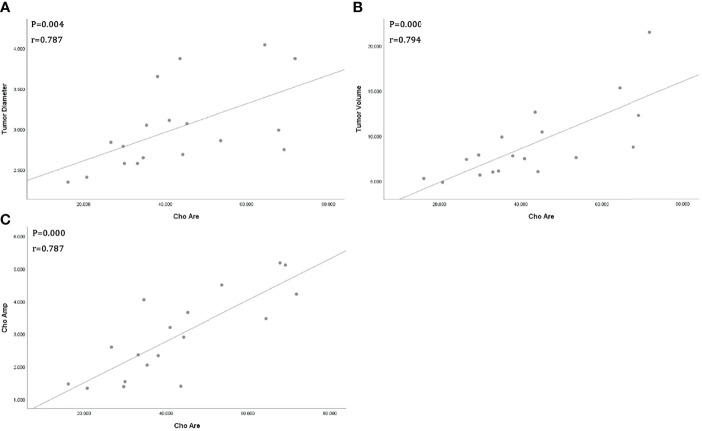
Scatter plot represent positive correlations between Choline (Cho) peak area and tumor diameter **(A)**, tumor volume **(B)**, and Cho peak amplitude **(C)**, respectively.

**Figure 3:**
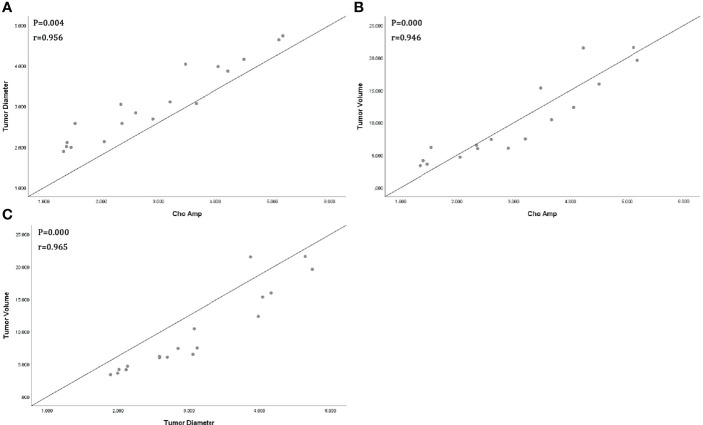
Scatter plot represents positive correlations between Choline (Cho) peak amplitude and tumor diameter **(A)**, tumor volume **(B)**, and positive correlation between tumor diameter and tumor volume **(C)**.

All tumor diameters were successfully measured in the MRI sequence, and the tumor volumes were calculated. The ICC consistent coefficients of tumor diameter and tumor volume were 1.000 (*p* < 0.001) and 1.000 (*p* < 0.001), respectively. The mean values and standard deviations of tumor diameter, tumor volume, Cho Are, and Cho Amp were 3.082 ± 0.940 cm, 9.779 ± 6.319 cm^3^, 42.461 ± 16.799, and 2.932 ± 1.305, respectively (**Table 1**). There were positive correlations between tumor diameter, tumor volume, and Cho Are and Cho Amp. As shown in **Figure 2**, Cho Are was highly positively correlated with tumor diameter and tumor volume, and the correlation coefficients r were 0.787 (*p* < 0.05) and 0.794 (*p* < 0.001), respectively. There was also a highly positive correlation between Cho Are and Cho Amp (r = 0.787, *p* < 0.001). As shown in **Figure 3**, Cho Amp was highly positively correlated with tumor diameter and tumor volume, and the correlation coefficients r were 0.956 (*p* < 0.05) and 0.946 (*p* < 0.001), respectively. There was also a highly positive correlation between tumor diameter and tumor volume (r = 0.965, *p* < 0.001).

**Table 1:** The mean values and standard deviations of tumor diameter, tumor volume, Cho Are, and Cho Amp in VX2 liver cancer (mean ± SD, n = 18).

Index	Tumor diameter (cm)	Tumor volumeTumor diameter (cm^3^)	Cho Are	Cho Amp
Mean ± SD	3.082 ± 0.940	9.799 ± 6.319	42.461 ± 16.799	2.932 ± 1.305

Cho Are, choline peak area; Cho Amp, choline peak amplitude.

### ROC Curve Analysis

**Figure 4:**
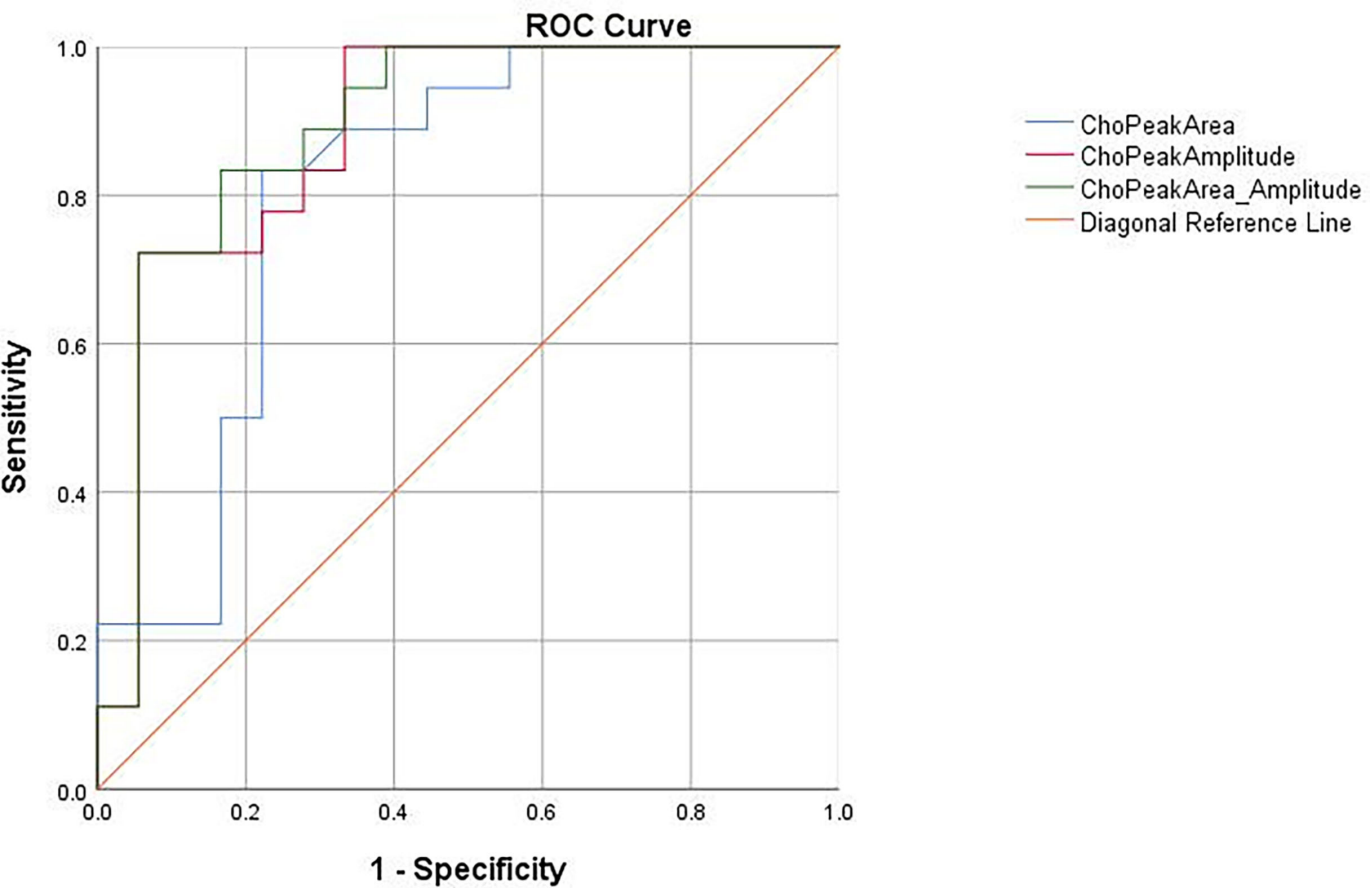
The receiver operating characteristic (ROC) curves for the diagnosis of liver cancer based on choline peak area (Cho Are), choline peak amplitude (Cho Amp), and choline peak area combined with choline peak amplitude (Cho Are_Amp).

In our study, Cho Are and Cho Amp were performed for ROC curve analysis. As shown in **Table 2** and **Figure 4**, the AUC of Cho Amp was 0.883, and the specificity and sensitivity were 0.944 and 0.722, respectively. The AUC of Cho Are was 0.807, and the specificity and sensitivity were 0.778 and 0.833, respectively. When Cho Are was combined with Cho Amp, the AUC of Cho Are_Amp was 0.892, and the specificity and sensitivity were 0.833 and 0.833, respectively.

**Table 2:** Diagnostic efficiency of Cho Are, Cho Amp, and Cho Are_Amp in VX2 liver cancer.

Index	AUC	*p*	95% CI	Sensitivity	Specificity
Cho Are	0.807	0.002	0.657–0.957	0.833	0.778
Cho Amp	0.883	0.000	0.766–0.999	0.722	0.944
Cho Are_Amp	0.892	0.000	0.780–1.000	0.833	0.833

Cho, choline; Cho Are, choline peak area; Cho Amp, choline peak amplitude; Cho Are_Amp, choline peak area combined with choline peak amplitude.

### Histology

**Figure 5:**
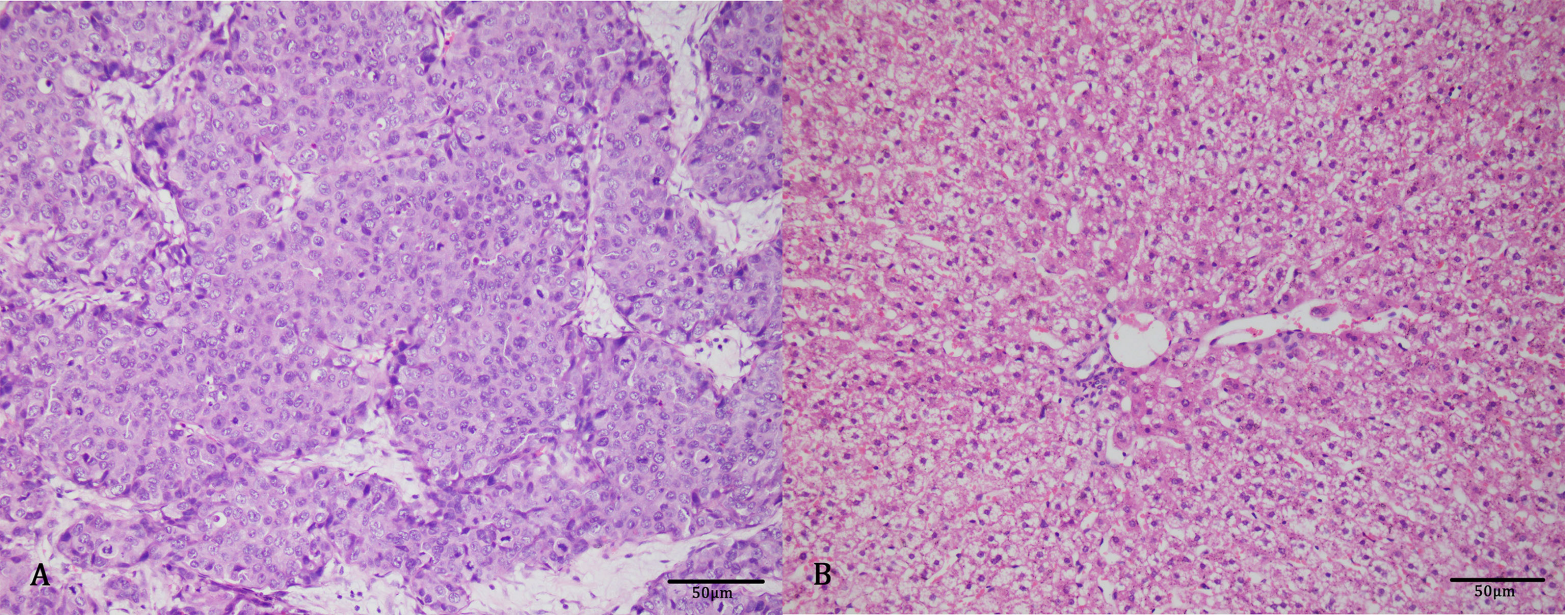
Hematoxylin and eosin (H&E) staining showed that the nucleus was large and deeply stained, the heterotypic mitotic phase was visible, and no obvious necrosis was found **(A)**. The structure of liver tissues was clear, and no obvious heterogeneity was found **(B)**.

H&E staining (**Figure 5A**) showed that the normal cell morphology disappeared and the tumor cell was not orderly arranged. The nuclei were large and dark stained, with atypia of the mitotic phase and no obvious necrotic cystic area. **Figure 5B** shows that the structure of hepatocytes was clear, the ratio of nucleus to cytoplasm was normal, and there was no obvious heterogeneity.

## Discussion

In this study, we concluded that Cho Amp and Cho Are_Amp of MRS harbored high diagnostic efficacy in early-stage rabbit VX2 liver cancer. Besides, Cho Are and Cho Amp were positively correlated with tumor size. Our results supported the application of these imaging indexes in early-stage liver cancer detection and extended the scope beyond a previous report with advanced stage only (14).

MRS remained the only non-invasive magnetic resonance technology to detect the metabolites of living tissue with quantitative and semi-quantitative properties (15). With the increase in magnetic field intensity, the signal-to-noise ratio of the static magnetic field changed with the chemical shift in a positive proportion. This allowed the quantitative measurement of MRS metabolites, such as peak amplitude and peak area (16–18). The accuracy relied on the stability of the spectral baseline, which could be negatively affected by respiration and intestinal disturbance (19, 20). Hence, most spectral studies were focused on organs with less mobility, and applications on liver were limited because of interferences mentioned above. To address this, intravenous anesthesia and respiratory gating technology were adopted to assure the stability of the spectral baseline so that all the rabbits successfully completed the scanning with distinguishable spectral lines.

Cho Are was widely reported to evaluate the change of metabolites in liver cancer (21, 22). However, Cho Are could be negatively disturbed by multiple factors causing inaccurate results. Poor magnetic field uniformity and motion artifacts may widen the metabolite peak and overall frequency shift, further resulting in the calculation mistake of the peak area. In contrast, we found that Cho Amp was more stable and had better diagnostic accuracy. This could be partially explained by the peak amplitude of the spectral line that was positively proportional to the metabolite concentration, less affected by shimming, and easier to observe. Therefore, Cho Amp was more accurate and objective to represent the content of compounds, consistent with a previous report in the head and neck (23). Interestingly, Cho Are_Amp had the highest diagnostic accuracy compared with each single parameter.

The Cho peak in MRS could indirectly reflect the change of choline compound content. Our study demonstrated that Cho Are showed a great positive correlation with both tumor diameter and volume, suggesting that choline compounds positively altered with tumor size. It may be attributed to the increased need of choline content in tumor proliferation, as well as the enhanced metabolism of phospholipids in the cell membrane (24). This observation was in line with previous findings (25). It was also worth noting that Voert and colleagues found that choline compounds were negatively correlated with tumor size (26), opposite to our findings. This might be explained by the tumor cells which were in a more advanced stage in their research compared to our early-stage neoplasm. Tumor differentiation and proliferation varied across different stages. Tumor in the advanced stage was accompanied by necrosis to a certain extent whose major component was lipid with reduced content of choline. Therefore, the choline peak may represent a downward trend. In contrast, the tumors in our subjects were all in the early stage and no necrosis was detected by MRI confirmed by subsequent pathological section. Additionally, there was a strong positive correlation between Cho Are and Cho Amp. Cho Amp was more sensitive with tumor diameter and volume change. Cho Amp may better display the changes of spectral Cho peak.

There are also some limitations in this study. Firstly, the single-voxel method was used in this study. Only the spectrum of a single part and a small number of tissues were obtained for analysis. MRS measurement could also be affected by other factors, including analysis software, operator experience, signal-to-noise ratio, and scanning time. Secondly, to ensure the stability and recognizability of the spectral line, a large voxel was set for collection, which may not be suitable for the small liver cancer less than 2 cm. Besides, the large number of monomeric spectral voxels may lead to low spatial resolution and reduced sensitivity to the change in metabolite content. Therefore, we cannot exclude the missing detection of small changes in choline content.

## Conclusion

The application of 3.0T magnetic resonance ^1^H-MRS was feasible in detecting early-stage rabbit VX2 liver cancer. Cho Amp exhibited better diagnostic accuracy compared with Cho Are. Cho Are_Amp harbored the highest diagnostic accuracy among all the three parameters. Cho Are and Cho Amp were highly positively correlated with each other, as well as the tumor volume and diameter. Therefore, Cho Amp and Cho Are_Amp of MRS may aid in diagnosing early-stage liver cancer.

## Data Availability Statement

The original contributions presented in the study are included in the article/supplementary material. Further inquiries can be directed to the corresponding author.

## Ethics Statement

The animal study was reviewed and approved by the Laboratory Animal Welfare and Ethics of the Third Military Medical University (No. SYXK-PLA-20120031).

## Author Contributions

DZ designed the study and revised the manuscript. RL conducted the experiments and drafted the manuscript. BZ and HX collected the data. ZT and LL analyzed the data. XL, JY, and CY performed the statistical analysis. All authors contributed to the article and approved the submitted version.

## Funding

The funding for this study was obtained from the Medical Research Program of the combination of Chongqing National Health Commission and Chongqing Science and Technology Bureau, China (No. 2021MSXM155), and the Medical Research Key Program of the combination of Chongqing National Health Commission and Chongqing Science and Technology Bureau, China (No. 2019ZDXM010).

## Conflict of Interest

The authors declare that the research was conducted in the absence of any commercial or financial relationships that could be construed as a potential conflict of interest.

The handling editor declared a shared parent affiliation with the authors DZ, RL, ZT, XL, LL, CY, HX, BZ, and JY at the time of review.

## Publisher’s Note

All claims expressed in this article are solely those of the authors and do not necessarily represent those of their affiliated organizations, or those of the publisher, the editors and the reviewers. Any product that may be evaluated in this article, or claim that may be made by its manufacturer, is not guaranteed or endorsed by the publisher.
